# The PI3K–NF-κB signal transduction pathway is involved in mediating the anti-inflammatory effect of IB-MECA in adjuvant-induced arthritis

**DOI:** 10.1186/ar1887

**Published:** 2006-01-13

**Authors:** Pnina Fishman, Sara Bar-Yehuda, Lea Madi, Lea Rath-Wolfson, Avivit Ochaion, Shira Cohen, Ehud Baharav

**Affiliations:** 1 Can-Fite BioPharma Ltd., Kiryat-Matalon, Petah-Tikva, Israel; 2 Felsenstein Medical Research Center, Rabin Medical Center, Sackler Faculty of Medicine Tel-Aviv University, Petach-Tikva, Israel; 3 Department of Pathology Rabin Medical Center, Sackler Faculty of Medicine Tel-Aviv University, Petach-Tikva, Israel

## Abstract

The anti-inflammatory effect of adenosine was previously found to be mediated via activation of the A_3 _adenosine receptor (A_3_AR). The aim of the present study was to decipher the molecular mechanism involved with the inhibitory effect of IB-MECA, an A_3_AR agonist, on adjuvant-induced arthritis.

The adjuvant-induced arthritis rats responded to IB-MECA treatment with a decrease in the clinical score and the pathological score of the disease. The response to IB-MECA was neutralized by the antagonist MRS 1220, confirming that the efficacy of the synthetic agonist was A_3_AR mediated.

The A_3_AR protein expression level was highly expressed in the synovia, in the peripheral blood mononuclear cells and in the drain lymph node (DLN) tissues of adjuvant-induced arthritis rats in comparison with naïve animals. Downregulation of A_3_AR expression was noted upon treatment with IB-MECA. Analysis of synovia and DLN protein extracts revealed a decreased expression level of PI3K, PKB/Akt, IKK, NF-κB and tumor necrosis factor alpha, known to affect survival and apoptosis of inflammatory cells, whereas the caspase-3 level was upregulated.

Taken together, high A_3_AR expression is found in the synovia, in the immune cells in the DLN and in peripheral blood mononuclear cells. IB-MECA, an orally bioavailable molecule, activates the A_3_AR, inducing receptor downregulation and the initiation of a molecular mechanism that involves de-regulation of the PI3K–NF-κB signaling pathway. As a result, a potent anti-inflammatory effect manifested in the improvement of the disease clinical score and pathological score occurs. The finding that the A_3_AR expression level in the peripheral blood mononuclear cells and in the DLN reflects the receptor status in the remote inflammatory site suggests use of the A_3_AR as a follow-up biomarker.

## Introduction

Considerable evidence has accumulated indicating that adenosine, through its receptors, plays an important role in limiting inflammation. Adenosine's anti-inflammatory effects are manifested by inhibition of tumor necrosis factor alpha (TNF-α), IL-1 and IL-6 production [1-3]. These responses have been shown *in vitro *in neutrophil and macrophage cell lines as well as in synoviocytes [4-7]. It is quite impossible to assess the effect of adenosine *in vivo *due to its rapid metabolization by adenosine deaminase. The involvement of adenosine in mediating the effect of several anti-inflammatory drugs such as aspirin, methotrexate and sulfasalazin has been described, supporting the role of adenosine in the regulation of the inflammatory process [8,9]. The dichotomy between the high adenosine levels in the inflamed tissues and the inability of adenosine to hamper the inflammatory process is explained by the increased adenosine deaminase level in this environment [10].

Recent studies suggested that the A_3 _adenosine receptor (A_3_AR) plays a major role in mediating the anti-inflammatory effect of adenosine. The highly selective A_3_AR agonist 1-deoxy-1-(6-{[(3-iodophenyl)methyl]amino}-9H-purine-9-yl)-*N*-methyl-β-d-ribofuranuronamide (IB-MECA) inhibited the production of TNF-α and MIP-1α *in vitro*, and prevented the development of collagen and adjuvant-induced arthritis (AIA) in experimental animal models [11,12]. Moreover, methotrexate was not efficacious in A_3_AR knockout mice in which inflammation was induced, thus confirming the role of adenosine and of the A_3_AR in the regulation of the anti-inflammatory response [13].

The A_3_AR belongs to the family of the Gi-protein-associated cell membrane receptors. Receptor activation leads to inhibition of adenylyl cyclase activity, inhibition of cAMP formation and inhibition of PKA expression, resulting in the initiation of various signaling pathways [14]. Our earlier studies showed that the A_3_AR is highly expressed in tumor cells. Receptor activation by IB-MECA inhibited the growth of melanoma, prostate carcinoma and colon carcinoma *in vitro *as well as in syngeneic and xenograft models *in vivo *[15-17]. The mechanistic pathway involved A_3_AR downregulation shortly after treatment, which subsequently induced a decrease in the expression of PKAc and PKB/Akt. The latter is known to control the NF-κB level by phosphorylating downstream proteins such as IKK and IκB, which in turn release NF-κB from its complex [15]. NF-κB then translocates to the nucleus where it induces the transcription of TNF-α and additional inflammatory proteins [18]. Apoptotic pathways are also known to be controlled downstream to PKB/Akt. Caspase-9 and caspase 3, which are downregulated upon PKB/Akt activation, fail to activate pathways leading to apoptosis [19].

One of the major mechanisms responsible for the development of arthritis is the upregulation of NF-κB that results in increased TNF-α levels. Moreover, the incapability of inflammatory cells to undergo apoptosis leads to their accumulation in the joints, thus maintaining the inflammatory process [19-21].

In the present study we show that the A_3_AR in AIA rats is highly expressed in the synovia, in peripheral blood mononuclear cells (PBMNC) and in lymph node cells. Upon IB-MECA treatment, the receptor is downregulated and modulation of the PKB/Akt–NF-κB signal transduction pathway takes place, resulting in amelioration of the inflammatory process.

## Materials and methods

### Reagents

The A_3_AR agonist IB-MECA was synthesized for Can-Fite BioPharma by Albany Molecular Research Inc. (Albany, NY, USA). MRS 1220, a highly selective A_3_AR antagonist, was purchased from RBI/Sigma (Natick, MA, USA). For both reagents, a stock solution of 10 mM was prepared in dimethyl sulfoxide and was further diluted in PBS.

Rabbit polyclonal antibodies against the rat A_3_AR and the signaling proteins PI3K, IKKα/β, were purchased from Santa Cruz Biotechnology Inc. (Santa Cruz, CA, USA). NF-κB, TNF-α and caspase-3 were purchased from CHEMICON International, Inc (Temecula, CA, USA). total and phosphospecific PKB/Akt (S473) were purchased from Cell Signaling Technology, Inc. Danvers, MA, USA)

### Experimental adjuvant-induced arthritis model

Female Lewis rats, aged 8–12 weeks were obtained from Harlan Laboratories (Jerusalem, Israel). Rats were maintained on a standardized pelleted diet and were supplied with tap water. Experiments were performed in accordance with the guidelines established by the Institutional Animal Care and Use Committee at Can-Fite BioPharma (Petach Tikva, Israel). The rats were injected subcutaneously at the tail base with 100 μl suspension composed of incomplete Freund's adjuvant with 10 mg/ml heat-killed *Mycobacterium tuberculosis *(Mt H37Ra; Difco, Detroit, MI, USA).

Each experimental group contained 10 animals. Treatment was initiated on day 14 after vaccination, when the clinical arthritis is apparent. IB-MECA (10 μg/kg) and the antagonist MRS 1220 (10 μg/kg) were orally administered by gavage, twice daily. MRS 1220 was administered 30 minutes before IB-MECA. The control group received vehicle only (dimethyl sulfoxide in a dilution corresponding to that of the drugs). Treatment was given for 14 days and animals were sacrificed on day 28, 2 hours after the last treatment.

The clinical disease activity score was assessed by inspecting the animals every second day for clinical arthritis. The scoring system ranged from 0 to 4 for each limb (0 = no arthritis; 1 = redness or swelling of one toe/finger joint; 2 = redness and swelling of more than one toe/finger joints; 3 = involvement of the ankle and tarsal-metatarsal joints; 4 = entire paw redness or swelling). The clinical score was calculated by adding the four individual legs' score. The inflammatory intensity was also determined in accordance with the increase in the rat hind paw's diameter, measured by caliper (Mitotoyo, Tokyo, Japan).

The histology score was assessed as follows. Animals were sacrificed on day 28. The legs were then removed up to knee level, fixed in 10% formaldehyde, were decalcified, dehydrated and paraffin-embedded, were cut into 4 μm sections and were stained with H & E.

The assessment of all pathologic findings were performed using semiquantitative grading scales of 0 to 4 for the following parameters: the extent of inflammatory cells' infiltration to the joint tissues; synovial lining cell hyperplasia; pannus formation; joint cartilage layers destruction. The bone damage and erosion score was graded from 0 to 5 (0 = normal; 1 = minimal loss of cortical bone at a few sites; 2 = mild loss of cortical trabecular bone; 3 = moderate loss of bone at many sites; 4 = marked loss of bone at many sites; 5 = marked loss of bone at many sites with fragmenting and full thickness penetration of inflammatory process or pannus into the cortical bone). The mean of all the histological parameter scores were designated the 'histology score'.

### Separation of synovial cells, PBMNC and lymph node cells

Synovial tissue was excised and cells were separated by incubating the synovial tissue in RPMI containing 1 mg/ml collagenase IV and 0.1 mg/ml DNase with a vigorous shaking at 37°C for 30 min. The supernatant containing the synovial cells was collected and the undigested tissue was re-extracted. The supernatants from all extractions were combined and cells were washed with PBS.

Regional lymph nodes were removed and cells were separated by mincing the tissue and disaggregating it through a needle of 22 G.

PBMNC from naïve rats, AIA rats and IB-MECA-treated rats were fractionated from heparinized blood using the Ficoll–Hypaque gradient.

### Western blot analysis

Western blot (WB) analyses of synovial cells, PBMNC and lymph node cells were carried out according to the following protocol. Samples were rinsed with ice-cold PBS and were transferred to ice-cold lysis buffer (TNN buffer, 50 mM Tris buffer [pH 7.5], 150 mM NaCl, NP 40). Cell debris was removed by centrifugation for 10 min at 7500 × *g*. Protein concentrations were determined using the Bio-Rad protein assay dye reagent. Equal amounts of protein (50 μg) were separated by SDS-PAGE, using 12% polyacrylamide gels. The resolved proteins were then electroblotted onto nitrocellulose membranes (Schleicher & Schuell, Keene, NH, USA). Membranes were blocked with 5% BSA and were incubated with the desired primary antibody (dilution 1:1000) for 24 hours at 4°C. Blots were then washed and incubated with a secondary antibody for 1 hour at room temperature. Bands were recorded using a BCIP/NBT color development kit (Promega, Madison, WI, USA). WBs were normalized against the housekeeping protein actin. Data presented in the different figures are representative of at least four different experiments.

### PKB/Akt activity assay

After protein isolation, 100 μg from each sample was removed for the PKB/Akt activity assay. This was carried out utilizing an Akt kinase assay kit (Cell Signaling Technology, Inc. Danvers, MA, USA), utilizing the GSK-3β fusion protein as a substrate. The activity was detected by WB analysis and the bands were recorded using the BCIP/NBT color development kit (Promega).

### Statistical analysis

Repeated-measurements general linear models analysis of variance (ANOVA) was performed for testing differences in the changes between baseline assessment (day 14) and post-baseline assessment (day 28) between the four study groups for the clinical score and for the paw thickness. All tests applied were two-tailed, and a *P *value of 5% or less was considered statistically significant. The data were analyzed using the SAS software (SAS Institute, Cary, NC, USA).

Repeated-measurements analysis using the Dunkan method was applied following the ANOVA analysis. Additional exclusive analysis was performed only for the two main time points (days 7 and 28) because this period is the most interesting for the study, as it reflects the changes at study termination. The student's *t *test for the WB analysis samples and the statistical significance were set at *P *< 0.05.

## Results

### IB-MECA inhibits the clinical and pathological manifestations of AIA

**Figure 1:**
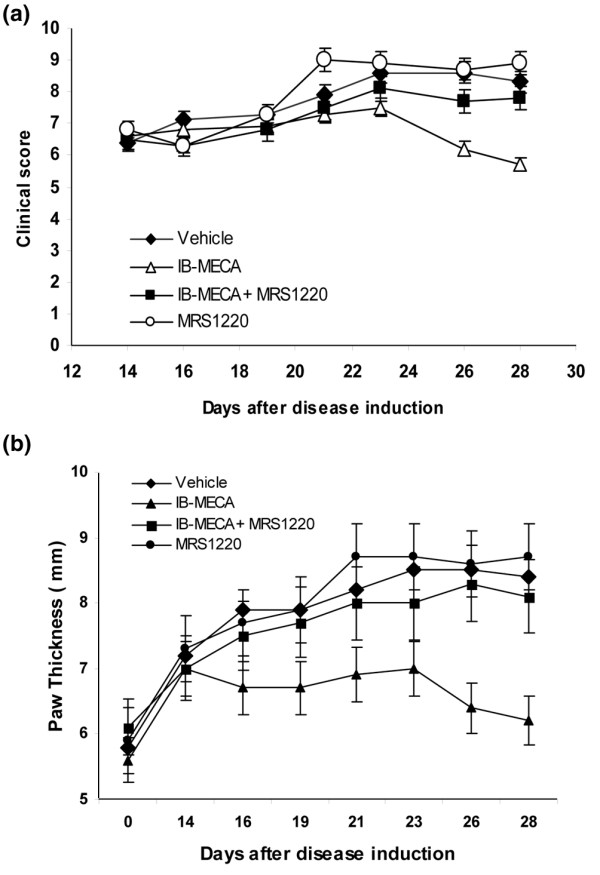
Effect of IB-MECA in the presence and absence of MRS 1220 on the clinical and pathological manifestations of adjuvant-induced arthritis. Rats were injected with an emulsion composed of incomplete Freund's adjuvant with 10 mg/ml heat-killed *Mycobacterium tuberculosis*. Treatment with IB-MECA (10 μg/kg) or the A_3 _adenosine receptor antagonist MRS 1220 (10 μg/kg), or a combination of both, was initiated on day 14. (**a**) Effect of IB-MECA on disease clinical score. (**b**) Effect of IB-MECA on paw thickness.

Approximately 21 days after immunization most of the vehicle-treated animals progressively developed arthritis. IB-MECA treatment (10 μg/kg orally twice daily, starting on day 14 after immunization) caused a significant decrease in disease severity as evaluated by the arthritis clinical score. Disease peaked on days 21–28 and the maximal effect of IB-MECA was seen on these days (Figure 1a). A similar pattern of disease activity was observed when paw thickness was measured (Figure 1b). ANOVA with repeated measurements was performed for testing differences in the parameters of clinical score and paw thickness between the four study groups: IB-MECA group, IB-MECA + MRS 1220 group, control group, and MRS 1220 group. The analysis was performed at two time points: day 7 (first measurement) and day 28 (last measurement). Statistically significant differences were found in the change between the two time points in the clinical score (*P *= 0.049) as well as in paw thickness (*P *= 0.001).

**Figure 2:**
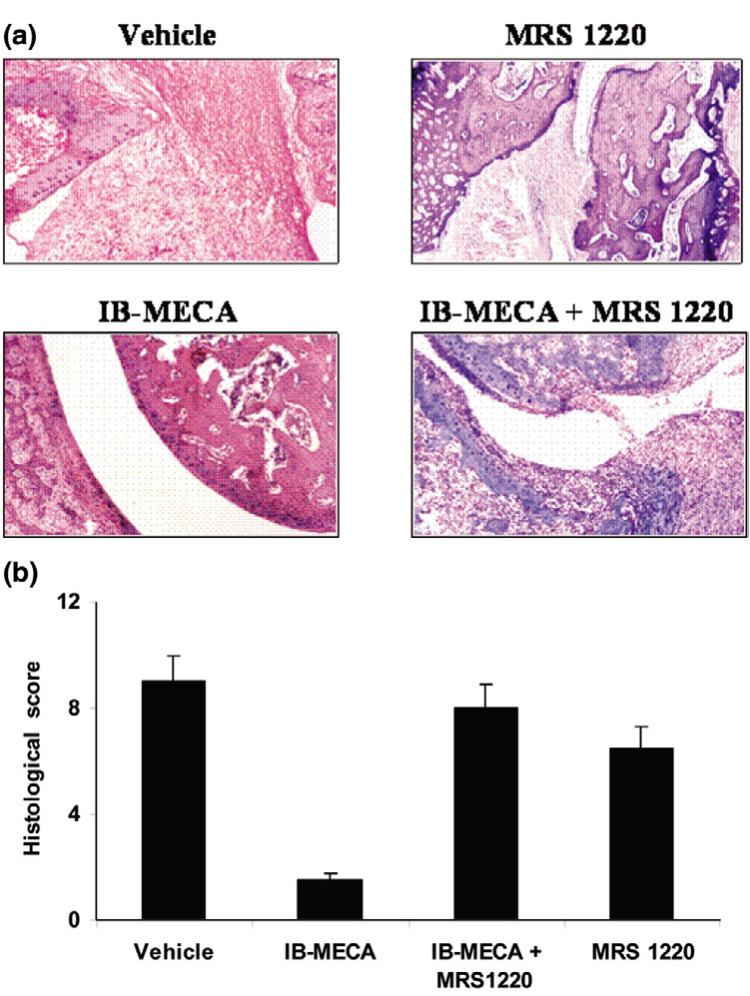
Effect of IB-MECA in the presence and absence of MRS 1220 on the clinical and pathological manifestations of adjuvant-induced arthritis. (**a**) Effect of IB-MECA on the pathological features of joint destruction in adjuvant arthritis. Shown are representative histology sections obtained after the rats were sacrificed on day 28. (**b**) Mean histology score.

Histological evaluation of the paws in the vehicle-treated arthritic animals revealed signs of severe arthritis with massive inflammatory cell infiltration, hyperplasia of the synovia, pannus formation, and bone and cartilage damage. IB-MECA suppressed these pathological and histological changes. No inflammatory infiltration or pannus formation were noted. The synovial membrane, bone and cartilage were preserved in the IB-MECA-treated rats (Figure 2a). The histological score was reduced from 9.1 ± 0.85 in the vehicle group to 2.5 ± 0.3 (*P *< 0.01) in the IB-MECA-treated group (Figure 2b). ANOVA for differences between the four study groups showed that the values measured in the IB-MECA group were statistically significantly lower than values measured in the other three groups (*P *< 0.001).

To test the specificity of the response to IB-MECA, rats were treated with the A_3_AR antagonist MRS 1220 alone or in combination with IB-MECA. MRS 1220 alone did not affect the clinical, pathological or histology score. When administered in combination with IB-MECA, it counteracted the IB-MECA's beneficial effect, resulting in a clinical score similar to that of the vehicle-treated group (Figure 1a,b). In addition, pathological manifestations and histology scores did not differ from the control group (Figure 2a,b). These findings support the assumption that the MRS 1220, an A_3_AR-specific antagonist, abrogated the therapeutic effect of IB-MECA.

### IB-MECA modulates the expression level of the A_3_AR and downstream key signaling proteins in the synovia, PBMNC and drain lymph node cells

**Figure 3:**
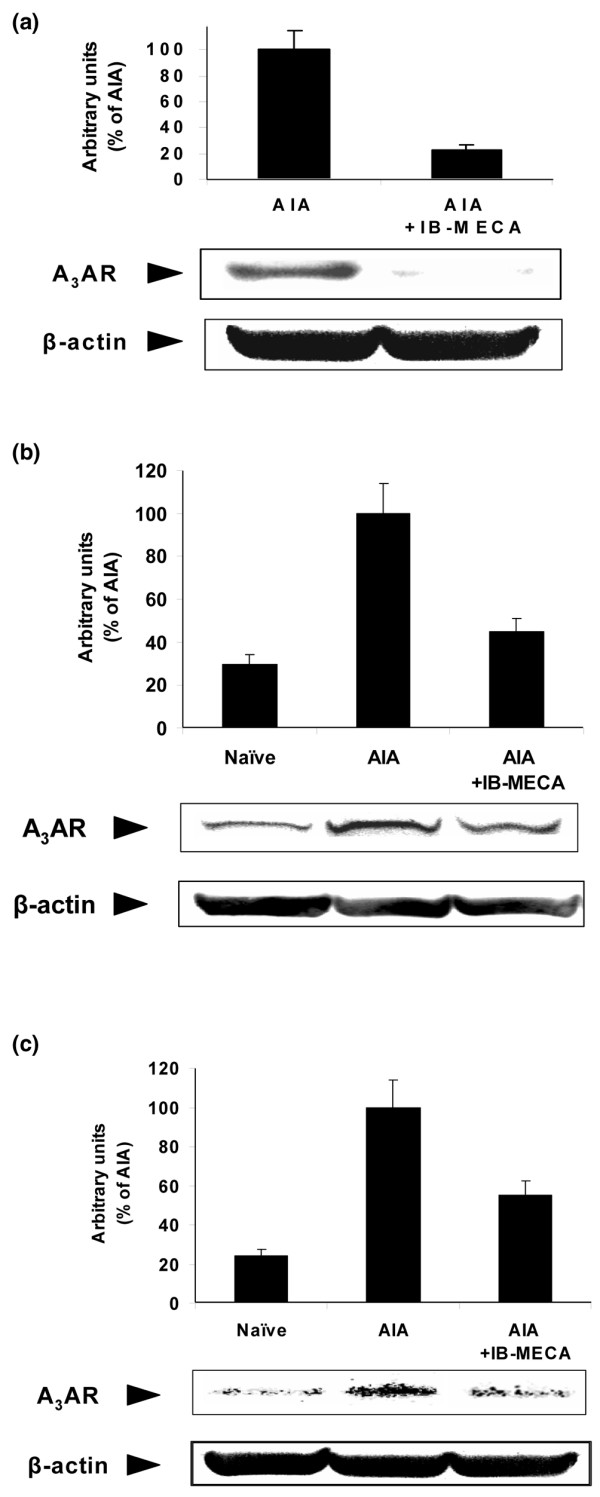
Western blot analysis of the A_3 _adenosine receptor (A_3_AR) in synovial cells, drain lymph node (DLN) cells and peripheral blood mononuclear cell (PBMNC) protein extracts. (**a**) A_3_AR expression in synovial tissue derived from untreated and IB-MECA-treated adjuvant-induced arthritis (AIA) rats. (**b**) A_3_AR expression in DLN cells from naïve, vehicle-treated, and IB-MECA-treated AIA rats. (**c**) PBMNC derived from naïve, vehicle-treated, and IB-MECA-treated AIA rats.

The A_3_AR was found to be highly expressed in the synovia, PBMNC and DL) cells derived from AIA rats in comparison with the corresponding naïve tissues (*P *< 0.01). Normal synovial tissue could not be evaluated for receptor expression since it is too thin to be excised. In IB-MECA-treated AIA rats, downregulation of the A_3_AR protein expression level was noted in all these cells (*P *< 0.01) (Figure 3a–c).

We then analyzed the effect of IB-MECA on the expression level of key signaling proteins downstream to the A_3_AR activation in synovial cells and DLN cell protein extracts.

**Figure 4:**
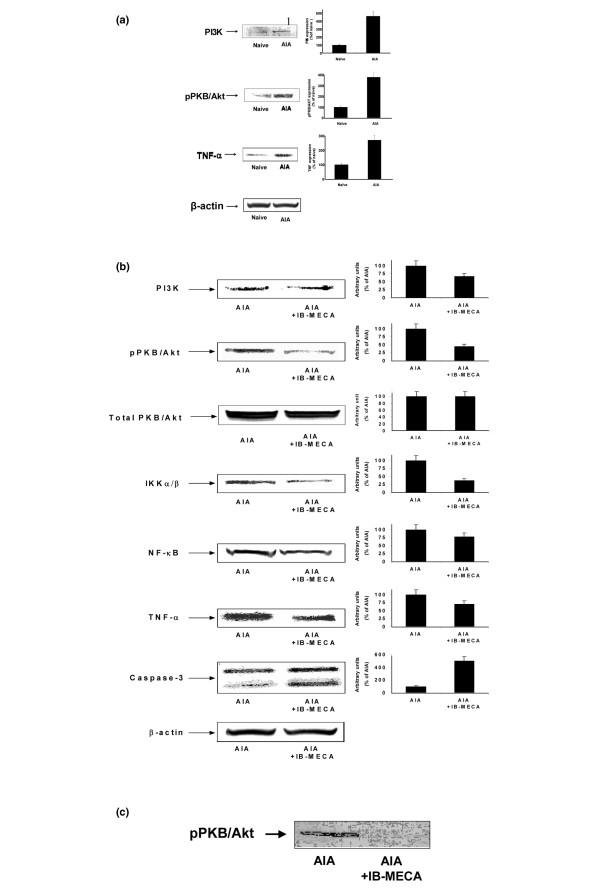
Western blot analysis of key signaling proteins downstream to A_3 _adenosine receptor (A_3_AR) activation in DLN extracts. (**a**) DLN cells derived from naïve and adjuvant-induced arthritis (AIA) animals. (**b**) DLN cells derived from AIA animals compared with AIA animals treated with IB-MECA. (**c**) PKB/Akt activity utilizing GSK-3β as a substrate in AIA animals in comparison with AIA animals treated with IB-MECA.

**Figure 5:**
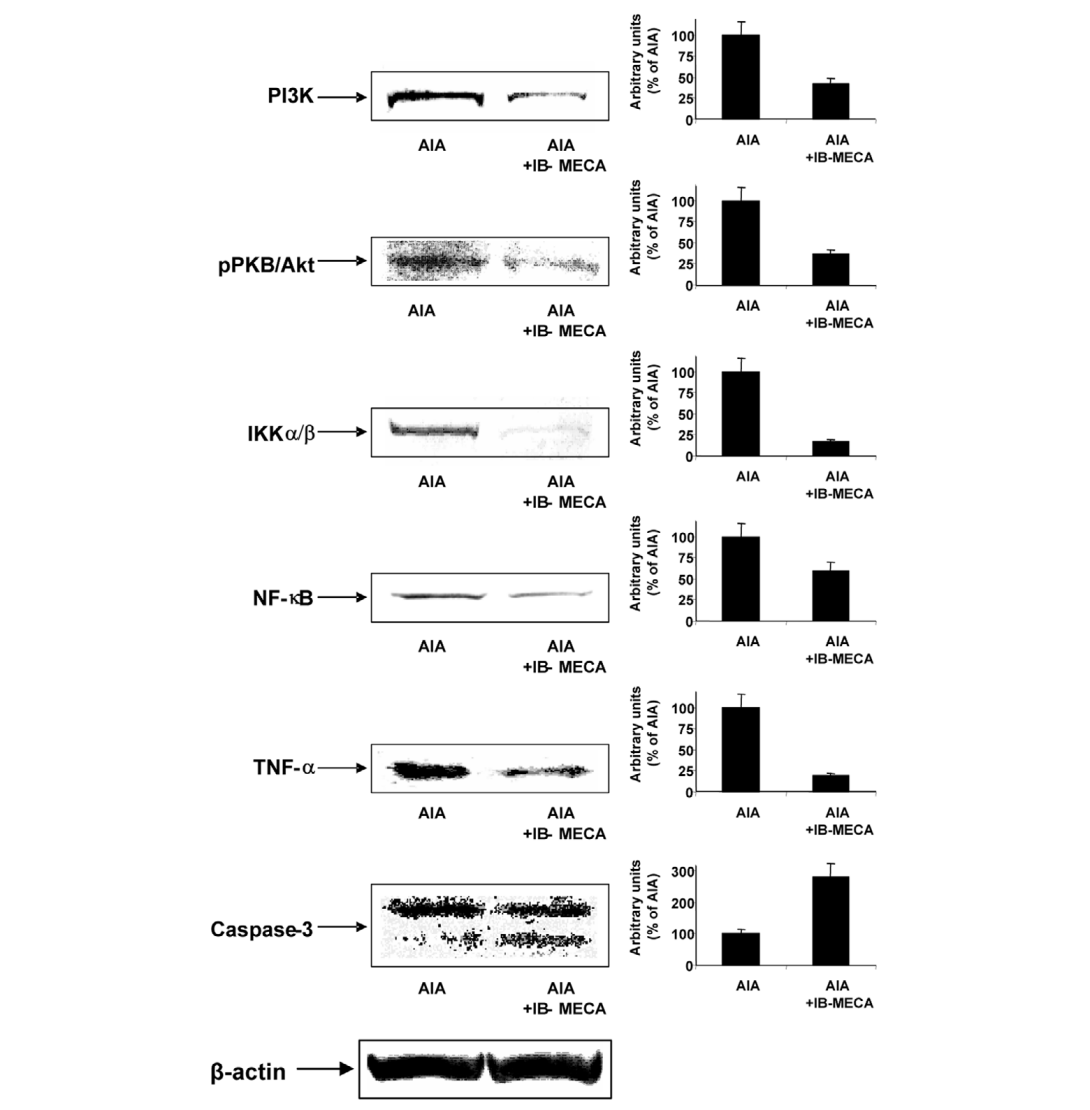
Effect of IB-MECA treatment on the expression level of key signaling proteins downstream to A_3 _adenosine receptor (A_3_AR) activation in synovia cells. The level of PI3K, pPKB/Akt, IKKα/β, NF-κB, TNF-α and caspase-3 was examined by western blot analysis. AIA, adjuvant-induced arthritis.

Induction of AIA induced upregulation in the expression level of various key signaling proteins such as PI3K, PKB/Akt (total and phosphorylated) and TNF-α, as measured in DLN protein extracts (Figure 4a). Upon IB-MECA treatment, the expression levels of PI3K, phosphorylated PKB/Akt, IKKα/β, NF-κB and TNF-α protein were downregulated (*P *< 0.05) (Figure 4b). We further confirmed the involvement of PKB/Akt in mediating the mechanism of action in DLN cell extracts. The PKB/Akt kinase activity was downregulated in the IB-MECA-treated group in comparison with the vehicle-treated IB-MECA + MRS 1220 group (Figure 4c). PI3K, PKB/Akt, IKKα/β, NF-κB and TNFα protein expression levels were also downregulated in synovia protein extracts (*P *< 0.01) (Figure 5). In both the synovia and DLN cells, an increase in the expression level of caspase-3 apoptotic proteins, known to be upregulated downstream to PKB/Akt inhibition, occurred (Figures 4b and 5) (*P *< 0.01).

## Discussion

In the present study we show that IB-MECA, a synthetic A_3_AR agonist, acts as an anti-inflammatory agent and ameliorates the development of AIA. IB-MECA inhibited the disease clinical score and the pathological manifestations of arthritis when given as a therapeutic agent. IB-MECA is considered one of the most highly selective A_3_AR agonists, with an affinity of 1.1 ± 0.3 nM to the rat A_3_AR [22].

In the present study we utilized two experimental approaches to show that the response to IB-MECA is specific toward the A_3_AR. The affinity value of IB-MECA to the A_3_AR is 50 times more than to the other adenosine receptors [23]. Thus, by treating the animals with a low dose (10 μg/kg) of IB-MECA, we are most probably targeting the A_3_AR and not other adenosine receptors. This assumption is based on human phase I studies in which we treated healthy subjects with 1 mg IB-MECA, resulting in a Cmax of 40 nM/ml [24]. Moreover, the selective antagonist MRS 1220 that was administered prior to IB-MECA treatment counteracted IB-MECA's effect, resulting in clinical and pathological scores similar to those of the control group.

An interesting finding of the present study was the high A_3_AR expression in the synovial cells, in PBMNC and in DLN cells derived from the AIA rats in comparison with naïve animals. The downregulation of A_3_AR protein expression, shortly after IB-MECA treatment, is typical of the G-protein coupled receptor phenomenon observed earlier by our group [17]. Similarly to the results of the present study, in tumor lesions derived from prostate or colon carcinoma-bearing mice, the A_3_AR was found to be highly expressed while downregulation was noted upon IB-MECA treatment receptor [16,25]. Further analysis of tumor cell growth regulatory proteins indicated that receptor downregulation was associated with a decrease in the level of PKB/Akt, β-catenin, NF-κB, cyclin D_1 _and c-Myc [15,26]. It was thus concluded that receptor downregulation represents receptor functionality and is accompanied by modulation of downstream cell growth regulatory proteins resulting in tumor growth inhibition.

In the present study, key signaling proteins in the synovial cells and DLN cells were also examined downstream to receptor activation. The expression levels of PI3K, PKB/Akt, IKKα/β, NF-κB and TNF-α were downregulated upon IB-MECA treatment. Earlier *in-vitro *studies also showed that A_3_AR activation in macrophages decreased the intracellular level of NF-κB, leading to a decrease in the transcription of TNF-α [27].

It has been documented that activated PKB/Akt is highly expressed in the synovial tissue of rheumatoid arthritis patients compared with its level in osteoarthritis patients [21]. PKB/Akt controls apoptotis via the modulation of downstream key signaling proteins that include NF-κB and caspases [28]. Indeed, IB-MECA treatment diminished the IKKα/β and NF-κB protein expression levels.

The extended lifespan of rheumatoid inflammatory cells such as neutrophils, lymphocytes, macrophages, fibroblasts and synoviocytes in the joints, and other inflammatory sites, is one of the hallmarks of rheumatoid arthritis [29,30]. One of the mechanisms that can contribute to this phenomenon is inhibition of apoptosis due to stimulation of the PI3K pathway, which leads to activation of PKB/Akt. The latter event phosphorylates several proteins such as GSK-3β, FKHR and BAD, which then fail to induce apoptosis. It may also prevent the expression of caspase-9 and caspase-3, proteins pivotal in the apoptotic cascade. Overexpression and activation of PKB/Akt have been defined as the main barrier of apoptosis in the inflamed rheumatoid arthritis tissues [31,32]. Interestingly, downregulation of phosphorylated PKB/Akt levels by wartmannin resulted in apoptosis of synoviocytes and macrophages in rheumatoid arthritis [33]. Similarly, our findings demonstrating PKB/Akt inhibition followed by an increase in caspase-3 level in the IB-MECA-treated animals supports the role of PKB/Akt in ameliorating the inflammatory process.

To the best of our knowledge, the present study is the first to show an *in-vivo *link between activation of the A_3_AR, inhibition of PKB/Akt and downstream signaling pathways leading to apoptosis in AIA.

The high receptor expression found in the immune system cells (PBMNC and DLN) reflects/mirrors the receptor status in the inflamed tissue. It was reported earlier that peripheral blood lymphocytes highly express the A_3_AR and reflect the high receptor expression in the tumor tissue in patients with colon carcinoma [34]. Other studies have shown that the expression and function of adenosine receptors may be regulated by proinflammatory cytokines that regulate receptor expression via a negative feedback loop [35,36]. It may thus be suggested that in the present study circulating levels of TNF-α induced A_3_AR upregulation in the synovia and in the PBMNC and DLN cells. Upon IB-MECA treatment and the downregulation of TNF-α levels, the receptor was also downregulated.

IB-MECA has been shown earlier to possess a potent anti-cancer effect against melanoma colon and prostate carcinoma. The treatment of autoimmune diseases with anti-cancer agents is a well-established concept and includes chemotherapy, cyclooxygenase-2 inhibitors, cytokines, antibodies against cytokines, and so on [37-39]. IB-MECA can thus be classified into the type of therapies that target mechanisms common to both diseases.

## Conclusion

It may be concluded that IB-MECA – a small, highly bioavailable molecule, found to be safe and well behaved in phase I human clinical trials [39] – may be a good drug candidate to combat the manifestations of rheumatoid arthritis. In addition, A_3_AR expression in the immune system cells may be suggested as a biomarker that reflects the receptor status in remote inflammatory sites.

## Abbreviations

A_3_AR = A_3 _adenosine receptor; AIA = adjuvant-induced arthritis; BSA = bovine serum albumin; DLN = drain lymph node; GSK-3β = glycogen synthase kinase-3β; H & E = hematoxylin and eosin; IB-MECA = 1-deoxy-1-(6-{[(3-iodophenyl)methyl]amino}-9H-purine-9-yl)-*N*-methyl-β-D-ribofuranuronamide; IKK = I Kappa Kinase; IL = interleukin; NF = nuclear factor; PBMNC = peripheral blood mononuclear cells; PBS = phosphate-buffered saline; PI3K = phospahtidylinositol-3 kinase; PKA = Protein Kinase A; PKB/Akt = Protein Kinase B; TNF-α = tumor necrosis factor alpha; WB = western blot.

## Competing interests

The authors declare that they have no competing interests.

## Authors' contributions

All authors read and approved the final manuscript.
